# Multi-dimensional classification of GABAergic interneurons with Bayesian network-modeled label uncertainty

**DOI:** 10.3389/fncom.2014.00150

**Published:** 2014-11-25

**Authors:** Bojan Mihaljević, Concha Bielza, Ruth Benavides-Piccione, Javier DeFelipe, Pedro Larrañaga

**Affiliations:** ^1^Computational Intelligence Group, Departamento de Inteligencia Artificial, Escuela Técnica Superior de Ingenieros Informáticos, Universidad Politécnica de MadridMadrid, Spain; ^2^Laboratorio Cajal de Circuitos Corticales, Centro de Tecnología Biomédica, Universidad Politécnica de MadridMadrid, Spain; ^3^Instituto Cajal, Consejo Superior de Investigaciones CientíficasMadrid, Spain

**Keywords:** probabilistic labels, consensus, distance-weighted k nearest neighbors, multiple annotators, neuronal morphology

## Abstract

Interneuron classification is an important and long-debated topic in neuroscience. A recent study provided a data set of digitally reconstructed interneurons classified by 42 leading neuroscientists according to a pragmatic classification scheme composed of five categorical variables, namely, of the interneuron type and four features of axonal morphology. From this data set we now learned a model which can classify interneurons, on the basis of their axonal morphometric parameters, into these five descriptive variables simultaneously. Because of differences in opinion among the neuroscientists, especially regarding neuronal type, for many interneurons we lacked a unique, agreed-upon classification, which we could use to guide model learning. Instead, we guided model learning with a probability distribution over the neuronal type and the axonal features, obtained, for each interneuron, from the neuroscientists' classification choices. We conveniently encoded such probability distributions with Bayesian networks, calling them *label Bayesian networks* (LBNs), and developed a method to predict them. This method predicts an LBN by forming a probabilistic consensus among the LBNs of the interneurons most similar to the one being classified. We used 18 axonal morphometric parameters as predictor variables, 13 of which we introduce in this paper as quantitative counterparts to the categorical axonal features. We were able to accurately predict interneuronal LBNs. Furthermore, when extracting crisp (i.e., non-probabilistic) predictions from the predicted LBNs, our method outperformed related work on interneuron classification. Our results indicate that our method is adequate for multi-dimensional classification of interneurons with probabilistic labels. Moreover, the introduced morphometric parameters are good predictors of interneuron type and the four features of axonal morphology and thus may serve as objective counterparts to the subjective, categorical axonal features.

## 1. Introduction

There are two main neuron subpopulations in the cerebral cortex: excitatory glutamatergic neurons, constituting approximately 80% of all cortical neurons, and inhibitory GABAergic interneurons, representing the remaining 20%. Although less numerous, GABAergic interneurons (for simplicity, interneurons), play multiple critical cortical functions and are highly heterogeneous with regards to their morphological, electrophysiological, and molecular properties (Ascoli et al., 2007). Neuroscientists consider that these differences indicate that various types of interneurons actually exist and that the differences among them are functionally relevant. Although many different classification schemes have been proposed so far (e.g., Fairén et al., 1992; Kawaguchi, 1993; Cauli et al., 1997; Somogyi et al., 1998; Gupta et al., 2000; Maccaferri and Lacaille, 2003), there is no universally accepted catalog of interneuron types (DeFelipe et al., 2013), making it hard to share and organize data and the knowledge derived from them. Ascoli et al. (2007) have identified a large set of morphological, electrophysiological, and molecular properties which can be used to distinguish among interneuron types. However, gathering such comprehensive data has considerable practical burdens (DeFelipe et al., 2013), making it hard to follow such a classification in practice.

Therefore, DeFelipe et al. (2013) proposed an alternative, pragmatic classification scheme, based on patterns of axonal arborization. The scheme classifies interneurons according to their type and four other features of axonal morphology. It contemplates ten types, most of them well established in literature, such as Martinotti and chandelier, and provides rather precise definitions of their axonal and dendritic morphology. The remaining axonal features are categorical properties such as axon's columnar and laminar reach (i.e., whether it is intra- or trans-columnar; intra- or trans-laminar)^1^. To assess the viability of this classification scheme, that is, whether it is useful for cataloging interneurons, DeFelipe et al. (2013) convened 42 leading neuroscientists to classify 320 interneurons. While the experts easily distinguished some of the neuronal types and the four remaining features, they found some types to be somewhat confusing.

Nonetheless, the data they gathered provides a basis for building an objective, automatized classifier, which would map quantitative neuronal properties to interneuron types and the categories of axonal features. Automatic classification of neurons has been mainly done in an unsupervised fashion (Jain, 2010), seeking to discover groups on the basis of quantitative properties alone (Cauli et al., 2000; Tsiola et al., 2003; Karagiannis et al., 2009; McGarry et al., 2010). However, the availability of expert-provided input on interneuron type membership and their axonal features allows us to learn a model in a supervised fashion (Duda et al., 2000), as done by, e.g., Marin et al. (2002) and Druckmann et al. (2013). When such supervision information is available, supervised learning can yield more accurate models than unsupervised learning (Guerra et al., 2011). In addition, a model obtained in this way can be used to replace experts, as it can, given an interneuron, automatically predict its properties (the type and axonal features).

Using the neuroscientists' classification choices as input for supervised classification is challenging due to the ambiguity in type membership and axonal features of the interneurons. While this ambiguity varied across our data, some interneurons were especially ambiguous: e.g., one was assigned to six different types, with at most 14 (out of 42) experts agreeing on one of these types. Previous efforts to predict the neuronal type and axonal features (DeFelipe et al., 2013; Mihaljević et al., 2014a,b) considered such majority choices as *ground truth*, i.e., as the true type and axonal features, and therefore, for each interneuron, disregarded the opinions of the disagreeing neuroscientists (with the majority for that interneuron). While Mihaljević et al. (2014b) only predicted the neuronal type, DeFelipe et al. (2013) and Mihaljević et al. (2014a) built an independent model for each axonal feature, although these features are complementary.

In this paper, we predict interneuron type and axonal features simultaneously, while accounting for class label ambiguity in a principled way. Namely, for each interneuron, we encode the neuroscientists' input with a joint probability distribution over the five class variables^2^. That is, we consider that each interneuron has a certain probability of belonging to each possible combination of the five axonal features. Assuming that all experts were equally good at classifying interneurons, these probabilities are given by the relative frequencies of such combinations in the expert-provided input. This way, we take the opinions of all annotator neuroscientists into account. Such probability distributions can be compactly encoded with Bayesian networks (Pearl, 1988; Koller and Friedman, 2009), given sufficient conditional independencies among the variables. We will therefore represent these joint probability distributions over class variables with Bayesian networks and call them *label Bayesian networks* (LBNs). As a first step in the present study, we will obtain LBNs from the experts' input; subsequently, we will train and evaluate our model using LBNs as input.

To the best of our knowledge, this is the first paper tackling multi-dimensional classification (i.e., with multiple class variables; Van Der Gaag and De Waal, 2006; Bielza et al., 2011) with probabilistic labels. Multi-dimensional classification is hard because of dependencies among class variables: ignoring them, by building a separate model for each variable, is suboptimal, while modeling them can result in data scarcity if there are more than a few class variables. Instead of identifying global dependencies among class variables, we predict the LBN of an interneuron by looking at the interneurons most similar to it (i.e., its neighbors in the space of predictor variables), following the lazy-learning *k*-nearest neighbors method (*k*-nn) (Fix and Hodges, 1989). Having found the neighbors of an interneuron, we predict its LBN by forming a consensus Bayesian network (e.g., Matzkevich and Abramson, 1992) among the neighbors' LBNs. In order to give more weight in the consensus distribution to the LBNs of the closer neighbors, we adapt the Bayesian network consensus method developed by Lopez-Cruz et al. (2014).

Note that our method takes LBNs, rather than the expert-provided labels, as input, thus abstracting away the annotators. In a similar real-world scenario, this might be useful for hiding the annotators' labels from the data analyst, for reasons such as confidentiality protection. Furthermore, an LBN could be obtained in multiple ways: by learning from data, eliciting from an expert, or combining expert knowledge and learning from data.

In order to predict the neuronal type and axonal features, we introduce 13 morphometric parameters of the axon to be used as predictor variables. We defined these parameters seeking to capture the concepts represented by the four axonal features (other than neuronal type) and implemented software that computes them from digital reconstructions of neuronal morphology. In addition, we used five other axonal morphometric parameters, computed with NeuroExplorer (Glaser and Glaser, 1990), which were already used as predictors of neuronal type by DeFelipe et al. (2013) and Mihaljević et al. (2014b). In total, we used 18 axonal morphometric parameters as predictor variables.

We found that our method accurately predicted the probability distributions encoded by the LBNs. Also, for comparison with previous work on interneuron classification, we assessed the prediction of the majority class labels, and found that we outperformed (DeFelipe et al., 2013) in per-class majority label accuracy.

The rest of this paper is structured as follows. Section 2 describes the data set, the interneuron nomenclature due to DeFelipe et al. (2013), the morphometric parameters, including the ones we introduce in this paper, and the extraction of LBNs from expert-provided labels; it also describes the proposed method—the distance-weighted consensus of *k* nearest Bayesian networks—, the related methods, the metrics for assessing our method's predictive performance, and, finally, specifies the experimental setting. We provide our results in Section 3, discuss them in Section 4, and conclude in Section 5.

## 2. Materials and methods

### 2.1. Neuronal reconstructions

We used neuronal reconstructions and expert neuroscientists' terminological choices that were gathered by DeFelipe et al. (2013). Of the 320 interneurons classified in that study, 241 were digitally reconstructed cells (retrieved by DeFelipe et al., 2013 from NeuroMorpho.Org, Ascoli et al., 2007), coming from different areas and layers of the cerebral cortex of the mouse, rat, and monkey. Forty of the reconstructions had one or multiple interrupted (i.e., with non-continuous tracing) axonal processes; when deemed feasible (36 cells), we unified the axonal processes using Neurolucida (MicroBrightField, Inc., Williston, VT, USA). We omitted the remaining four cells from our study, reducing our data sample to 237 cells.

### 2.2. Axonal feature-based nomenclature

DeFelipe et al. (2013) asked 42 expert neuroscientists to classify the above-described interneurons according to the interneuron nomenclature they proposed. The nomenclature consists of six categorical features of axonal arborization. The features' categories are the following:

1. Axonal feature 1 (*C*_1_): intralaminar and transla-minar
2. Axonal feature 2 (*C*_2_): intracolumnar and trans-columnar
3. Axonal feature 3 (*C*_3_): centered and displaced
4. Axonal feature 4 (*C*_4_): ascending, descending, both, and no
5. Axonal feature 5 (*C*_5_): arcade (AR), Cajal-Retzius (CR), chandelier (CH), common basket (CB), common type (CT), horse-tail (HT), large basket (LB), Martinotti (MA), neurogliaform (NG), and other (OT)
6. Axonal feature 6 (*C*_6_): characterized and unchar-acterized

**Figure 1:**
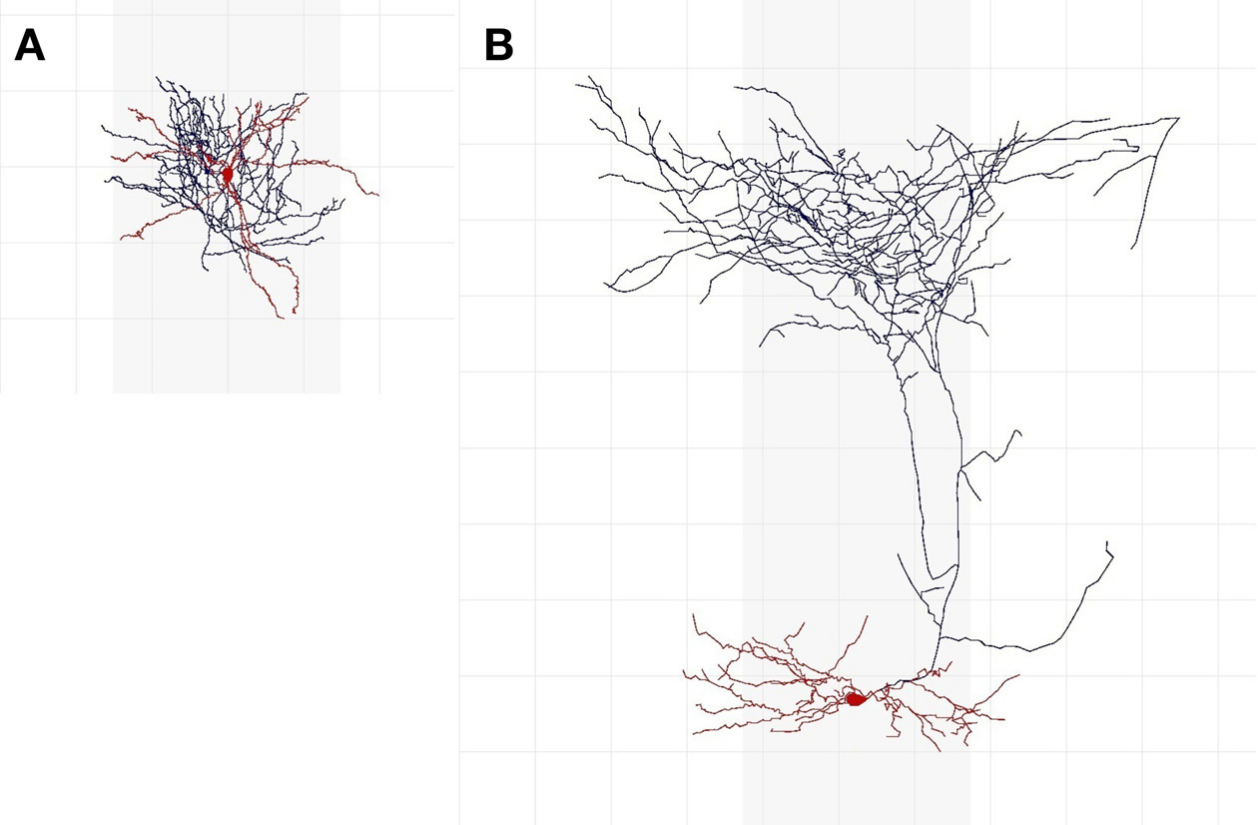
**Examples of interneurons of different types and axonal features. (A)** Is an intralaminar, intracolumnar, centered, and no cell, according to 37 (out of 42) experts. Most of its axon (shown in blue) is at less than 200 μm from the soma (shown in red; the grid lines are separated by 100 μm) and thus appears to be mainly located in soma's cortical layer; it is within soma's cortical column (the gray vertical shadows depict a 300 μm-wide cortical column); and it seems to be centered around the dendritic arbor (also shown in red). It is NG according to 18 experts, CB according to 17 experts, CT according to 3 experts, and OT and AR according to one expert each. **(B)** Is a translaminar, transcolumnar, displaced, and ascending cell according to 39 experts. Its axon reaches around 800 μm above soma (i.e., it seems to extend to another layer); a significant portion of its axon is outside of soma's cortical column; its dendrites are not in the center of the axonal arborization; and its axon is predominantly above the soma. According to 34 experts, this is a MA cell.

Cells whose axon is predominantly in soma's cortical layer are intarlaminar in *C*_1_; the rest are translaminar. Similarly, regarding *C*_2_, interneurons with the axon predominantly in soma's cortical column are intracolumnar; the rest are transcolumnar. A cell whose dendritic arbor is mainly located in the center of the axonal arborization is centered (*C*_3_); otherwise it is displaced. *C*_4_ further distinguishes between translaminar (*C*_1_) and displaced (*C*_3_) cells: cells with an axon mainly ascending toward the cortical surface are ascending, cells with an axon mainly descending toward the white matter are descending, whereas those with both ascending and descending arbors are termed both. To those cells that were not translaminar (*C*_1_) and displaced (*C*_3_) we assigned no in *C*_4_ (this category was not contemplated in the original nomenclature). Class *C*_5_ is the interneuron type. A cell is uncharacterized in *C*_6_ if it is not suitable for characterization according to features *C*_1_–*C*_5_, due to, e.g., insufficient reconstruction; otherwise, a cell is characterized. An expert who considered that a neuron was uncharacterized did not categorize it according to features *C*_1_–*C*_5_. Figure 1 shows two interneurons characterized according to axonal features *C*_1_–*C*_5_.

### 2.3. Predictor variables

We used 18 parameters of axonal morphology as predictor variables. Five of these parameters were computed with NeuroExplorer and were already used to predict interneuron types by DeFelipe et al. (2013) and Mihaljević et al. (2014b). In addition, we introduce 13 parameters of axonal morphology, seeking to the capture the concepts represented by axonal features *C*_1_–*C*_4_. We computed these parameters from 3D interneuron reconstructions files in Neurolucida's ASCII (^*^.asc) format.

The five parameters we computed with NeuroExplorer are:

1. *X*_1_: 2D convex hull perimeter (in *Z* projection).
2. *X*_2_: Axon length.
3. *X*_3_: Axon length at less than 150 μm from the soma.
4. *X*_4_: Axon length at more than 150 and less than 300 μm from the soma.
5. *X*_5_: Axon length at more than 300 μm from the soma.

Parameters *X*_3_–*X*_5_ are meant to measure axonal arborization with respect to the cortical column. Namely, parameter *X*_3_ approximates arborization length within a (300 μm wide) cortical column (at less than 150 μm from the soma); *X*_4_ approximates the length outside but not far from the column (more than 150 and less than 300 μm from the soma); and *X*_5_ approximates axonal length far from the column (more than 300 μm from the soma). *X*_1_ and *X*_2_ were used by DeFelipe et al. (2013) while Mihaljević et al. (2014a) used *X*_3_–*X*_5_ as predictor variables.

We introduce the following axonal morphometric parameters:

1. *X*_6_: Axon length within soma's layer.
2. *X*_7_: Axon length outside soma's layer.
3. *X*_8_: Proportion of axon length contained within soma's layer, $\frac{X_{6}}{X_{6}+X_{7}}$.
4. *X*_9_: Axon length within soma's cortical column.
5. *X*_10_: Axon length outside soma's cortical column.
6. *X*_11_: Proportion of axon length within soma's cortical column, $\frac{X_{9}}{X_{9}+X_{10}}$.
7. *X*_12_: Distance, in dimensions *X* and *Y*, from axon's centroid to the soma.
8. *X*_13_: Distance from the centroid of the above-the-soma part of the axon to the soma.
9. *X*_14_: Distance from the centroid of the below-the-soma part of the axon to the soma.
10. *X*_15_: Proportion of distances *X*_13_ and *X*_14_, $\frac{X_{13}}{X_{13}+X_{14}}$.
11. *X*_16_: Axon length above the soma.
12. *X*_17_: Axon length below the soma.
13. *X*_18_: Proportion of axon length above soma, $\frac{X_{16}}{X_{16}+X_{17}}$.

We computed these parameters following assumptions made by DeFelipe et al. (2013), namely: (a) cortical layer thickness is (roughly) determined by species and cortical area (see following paragraphs for details); and (b) the cortical column is a cylinder whose axis passes through the soma and has a diameter of 300 μm. We measured the distance to soma as the distance to soma's centroid. We computed a centroid of a set of points (e.g., of all the points comprising the reconstructed axon) by averaging those points.

When computing parameters *X*_6_ and *X*_7_ we looked up the approximate layer thickness according to the neuron's species and cortical area. DeFelipe et al. (2013) defined an approximate layer thickness for every species/area/layer combination present in their data, and provided it as additional information for experts who classified the interneurons. This information can be accessed at http://cajalbbp.cesvima.upm.es/gardenerclassification/. DeFelipe et al. (2013) specified the approximate thickness in the form of an interval—e.g., stating that layer II/III of the mouse's visual cortex is 200–300 μm thick—; we used the interval's midpoint (250 μm for the previous example) as an estimate of layer thickness. Also, we assumed that a soma is equidistant from the top and bottom confines of the layer (i.e., a 250 μm thick layer reaches 125 μm above and 125 μm below the soma).

For 16 mouse interneurons, seven of them from the somatosensory and nine from the visual cortex, the cortical layer was not provided. In order to compute variables *X*_6_ and *X*_7_ for these cells, we assumed them to belong to a hypothetical “average layer” for which we assumed a 197 μm thickness in the visual cortex and a 237 μm thickness in the somatosensory cortex. Although only an approximation, we consider this a more informed approximation to the “true” values of these variables than one that could be performed by a distance-computing rule (see Subsections 2.6 and 2.8) if we had left these values unspecified.

### 2.4. Data selection and summary

Axonal feature *C*_6_ is not a “proper” morphological feature but more of a “filter feature” which indicates whether the remaining axonal features can be reliably identified given a reconstructed interneuron. We therefore omitted *C*_6_ from consideration in this paper. Consequently, we removed from our data set 11 interneurons considered as uncharacterized by a majority (i.e., at least 21) of neuroscientists, considering that these interneurons cannot be reliably classified according to *C*_1_–*C*_5_, thereby reducing our data sample to 226 interneurons.

Thus, we have *N* = 226 interneurons, each of them quantified by a vector **X** of *m* = 18 real-valued predictor variables (i.e., **x** ∈ ℝ^18^). We also have *d* = 5 discrete class (i.e., target) variables **C** = (*C*_1_,…,*C*_5_), with **c** ∈ Ω_*C*_1__ × … × Ω_*C*_5__. Each interneuron, **x**^(*j*)^, is associated with a *N*_*j*_ × 5 (*N*_*j*_ ≤ 42) matrix 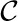
^(*j*)^ in which each row is an observation of **C** due to one annotator neuroscientist, i.e., 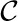
^(*j*)^_*i*,*a*_ is the label for class variable *C*_*i*_ assigned to interneuron **x**^(*j*)^ by expert neuroscientist *a*^3^.

Nonetheless, instead of the provided multi-annotator label matrices 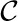, we require each interneuron to be associated with an LBN in order to apply our method. We obtained LBNs using standard procedures for LBNs from data, and then learned and evaluated our model using these LBNs as input, omitting 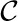 from further consideration (see the next subsection).

### 2.5. From multi-annotator labels to label Bayesian networks

Prior to applying our method, we learned LBNs from multi-annotator class label matrices 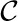.

An LBN is a Bayesian network over the class variables **C**. A Bayesian network (Pearl, 1988; Koller and Friedman, 2009) 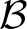 is a pair 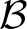 = (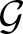,Θ) where 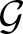, the structure of the network, is a directed acyclic graph whose vertices correspond to the class variables **C** and its arcs encode the conditional independencies in the joint distribution over **C**, while Θ are the parameters of the conditional probability distributions that the joint distribution is factorized into.

Learning a Bayesian network 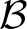 from data consists in two steps: learning network structure, 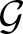 (i.e., the conditional independencies it encodes), and, having obtained the structure, learning its parameters (Neapolitan, 2004; Koller and Friedman, 2009). While the second step is generally straightforward, many methods exist for performing the first step. We applied a method belonging to the well-known family of search+score structure learning methods (see Subsection 2.8).

We wanted the learned LBNs to be similar to the actual empirical probabilities observed in the class label matrices, 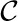. In other words, we wanted the probability distribution factorized by an LBN, *p*_
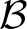
^(*j*)^_, to be similar to the empirical distribution, *p*_ϵ^(*j*)^_—the relative frequency of each possible state of **C** in 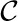
^(*j*)^. We used this similarity as criterion for selecting the network learning method (see Subsection 2.8) and measured it with Jensen-Shannon divergence (see Subsection 2.9.1).

**Figure 2:**
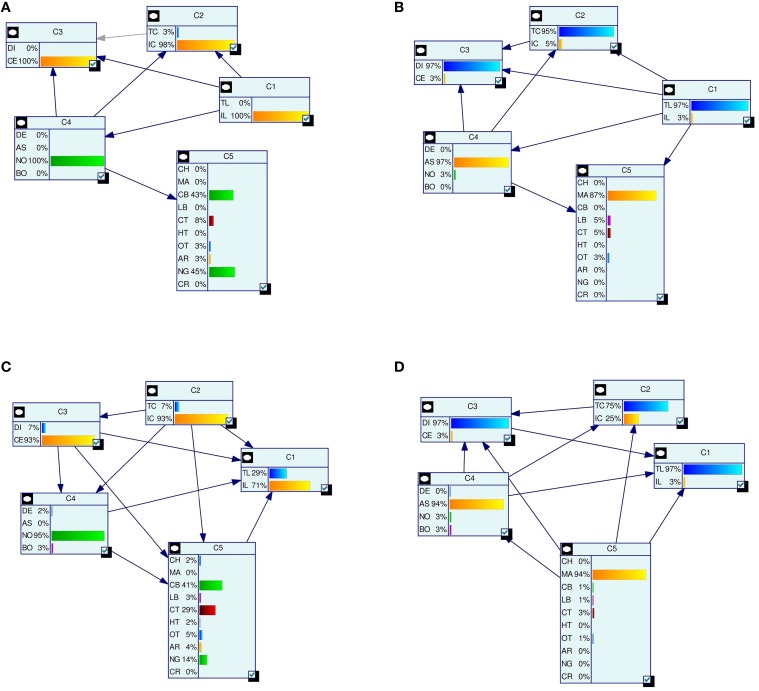
**Examples of true (A,B) and predicted (C,D) label Bayesian networks (LBNs) for neurons shown in Figure 1**. The leftmost networks **(A,C)** correspond to interneuron **(A)** in Figure 1 whereas the right-hand ones **(B,D)** correspond to neuron **(B)** in Figure 1. The Bayesian networks are depicted with their nodes (shown as rectangles), arcs, and each node's marginal probability distribution. The predicted distributions are similar to the true ones for many nodes—e.g., 93 vs. 98% for IC (node *C*_2_) for interneuron **(A)**. Some marginal probabilities do differ, such as that of the NG type for neuron **(A)**—14% predicted vs. 45% true; a lot of its probability mass was assigned to the more numerous CT type.

Finally, having learned the LBNs, our final data set was 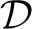 = {(**x**^(*j*)^, 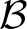
^(*j*)^)}^*N*^_*j* = 1_. Figure 2 depicts the LBNs for interneurons shown in Figure 1, along with the predicted LBNs for those interneurons.

### 2.6. Multi-dimensional classification with label Bayesian networks

Recall that we have *m* predictor variables **X**, with **x** ∈ ℝ^*m*^, that describe the domain under study, and *d* discrete class (or target) variables **C**, with **c** ∈ Ω_*C*_1__ × … × Ω_*C*_*d*__, that we wish to predict on the basis of observations of **X**. We observe a data set, 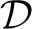 = {(**x**^(*j*)^, 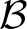
^(*j*)^)}^*N*^_*j* = 1_, where 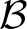 is a label Bayesian network encoding a joint probability distribution over the multi-dimensional class variable **C**.

**Figure 3:**
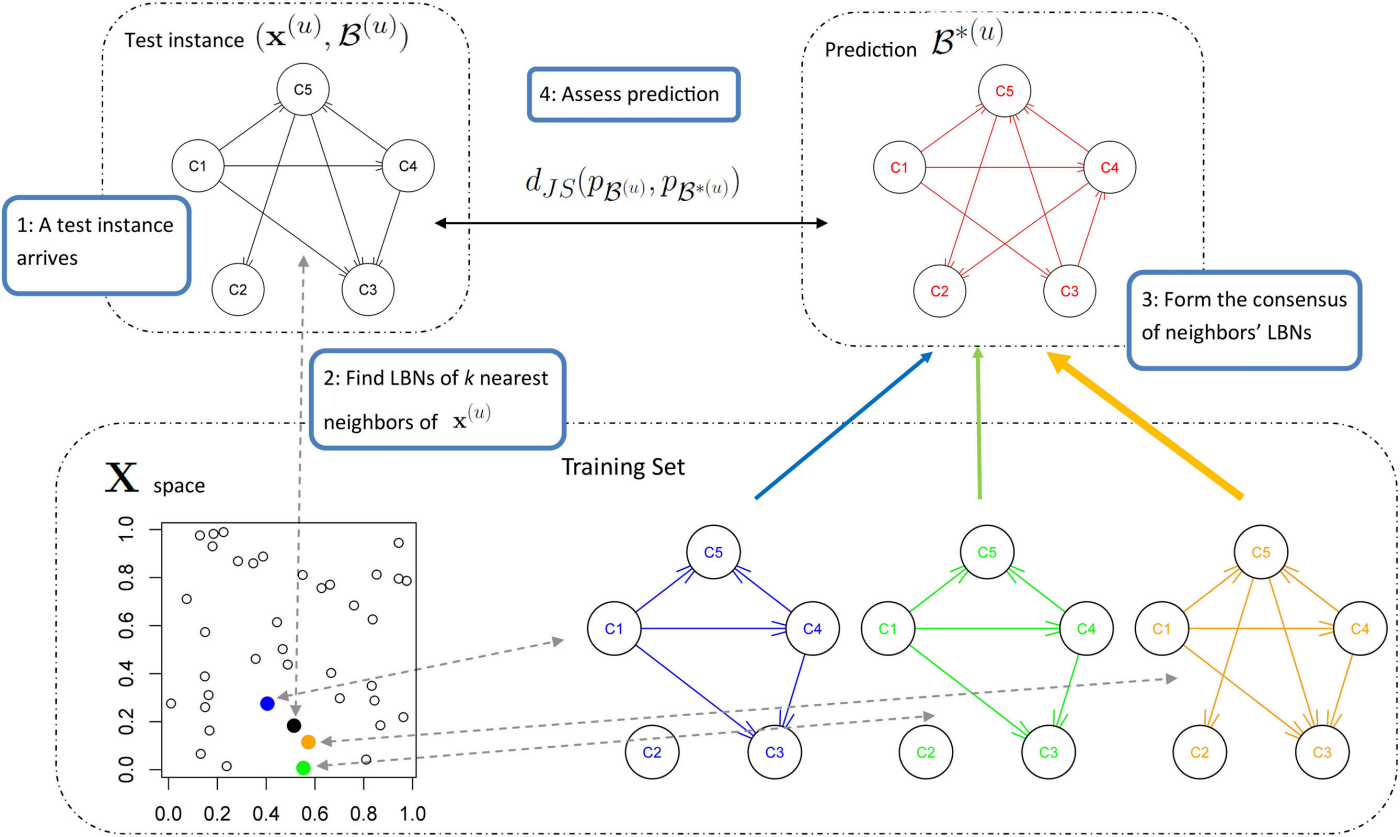
**A schematic representation of multi-dimensional classification with label Bayesian networks (LBNs)**. The figure depicts the assessment of our method's predictive performance. First (step 1; upper left), an instance **x**^(*u*)^ with LBN 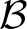
^(*u*)^ is retrieved from the test set. Then (step 2; lower part), we identify *k* (*k* = 3 in this example) nearest neighbors of **x**^(*u*)^ and record their distances to **x**^(*u*)^; the blue, green, and orange Bayesian networks (lower right) depict the LBNs of the three nearest neighbors of **x**^(*u*)^. Then (step 3; upper right), we obtain the predicted LBN, 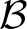
^*(*u*)^, by forming a consensus Bayesian network from the LBNs of the three nearest neighbors. Here, a thicker arrow suggests more weight of that neighbor's LBN in the consensus: the orange arrow is thicker than the blue and green arrows (orange is the closest neighbor of **x**^(*u*)^, see lower left). Finally (step four; upper middle), we compare true and predicted probability distributions, *p*_
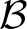
^(*u*)^_ and p_
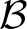
^*(*u*)^_, with Jensen-Shannon divergence.

We predict the LBN of an unseen instance **x**^(*u*)^ by forming a consensus Bayesian network among the LBNs of its *k* nearest neighbors (1 ≤ *k* < *N*) in the space of predictor variables. We form the consensus by adapting the method developed by Lopez-Cruz et al. (2014) to weigh the effect of each neighbor's LBN in proportion to that neighbor's relative closeness to **x**^(*u*)^. Figure 3 summarizes our approach.

*k* nearest neighbors (*k*-nn; Fix and Hodges, 1989) is an instance-based (i.e., model-less) classifier, popular in uni-dimensional classification (Duda et al., 2000). It classifies a data instance **x**^(*u*)^ by identifying its *k* nearest neighbors in the predictor space, according to some distance measure—a common choice is the Euclidean distance— and choosing the majority from the neighbors' labels.

#### 2.6.1. Distance-weighted consensus of Bayesian networks

Combining multiple Bayesian networks into a consensus Bayesian network is a recurring topic of interest. The standard methods for combining the parameters of a joint distribution, disregarding its underlying graphical structure (i.e., the conditional independencies), can yield undesirable results: for example, combining distributions with identical structures may render a consensus distribution with a different structure (Pennock and Wellman, 1999). It is therefore common to first combine network structures (e.g., Matzkevich and Abramson, 1992; Pennock and Wellman, 1999; Del Sagrado and Moral, 2003; Peña, 2011) and combine the parameters afterwards (e.g., Pennock and Wellman, 1999; Etminani et al., 2013). The cited structure-combining methods produce distributions which only contain independencies that are common to all networks, rendering them too complex (i.e., having too many parameters) to be useful in practice.

An alternative is to draw samples from the different Bayesian networks and learn the consensus network from the generated data, using standard methods for learning Bayesian networks from data (Neapolitan, 2004; Koller and Friedman, 2009), as proposed by Lopez-Cruz et al. (2014). Lopez-Cruz et al. (2014) weighted the influence of each Bayesian network on the consensus by sampling from it a number of instances proportional to its weight. We can readily adapt this method to weigh the effect of neighbors' label Bayesian networks in proportion to the their closeness to the instance being classified, **x**^(*u*)^, by defining an appropriate weighting function. Before defining the weighting function, let us state the setting more formally.

We want to generate a database 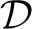
_*u*_ by sampling from *k* Bayesian networks {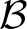
^(*j*)^}^*k*^_*j* = 1_ associated to *k* instances at distances *d*_1_, …, *d*_*k*_ from the unseen instance **x**^(*u*)^; from this database, 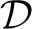
_*u*_, we will learn the consensus Bayesian network, 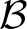
^*(*u*)^. We want the number of samples in 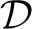
_*u*_ that are drawn from 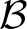
^(*j*)^ to be proportional to the how close **x**^(*j*)^ is to **x**^(*u*)^. We measure closeness as relative to the remaining *k* − 1 neighboring instances. Thus, if *M* is the desired size of 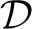
_*u*_ and **w** = (*w*_1_, …, *w*_*k*_) the weights assigned to the *k* Bayesian networks (with $\sum_{j\;=\;1}^{k}w_{j}=1$ and *w* ≥ 0), the number of samples from 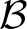
^(*j*)^, *M*_*j*_, is then *w*_*j*_ × *M*. We compute the weights as

$$w_{j}=\frac{(\sum_{i=1}^{k}d_{i})-d_{j}}{\left(k-1)(\sum_{i=1}^{k}d_{i}\right)}.$$

### 2.7. Related methods

#### 2.7.1. Multiple annotators

A setting similar to ours, where many annotators provide class labels, occurs when *learning from a crowd* of annotators (Snow et al., 2008; Sorokin and Forsyth, 2008; Raykar et al., 2010; Welinder et al., 2010; Raykar and Yu, 2012). Yet, the crowd may include annotators of different skills and therefore learning a classifier involves estimating the ground truth label from the possibly noisy ones. Methods such as those due to Dawid and Skene (1979); Whitehill et al. (2009); Raykar et al. (2010); Welinder et al. (2010); Raykar and Yu (2012) aim to detect the less reliable annotators and decrease their influence on the ground truth estimate. In our case, however, all annotators are domain experts; furthermore, there is currently no better approximation to ground truth than the opinions of this group of leading experts, as there is no unequivocal or objective way of determining it^4^. We thus consider that every expert's opinion is equally valid and that interneuron type membership and axonal features are uncertain whenever the experts do not completely agree. This allows us to represent interneuron type membership and axonal features of an interneuron with a joint probability distribution over these five class variables.

#### 2.7.2. Probabilistic labels

Probabilistic labels have already been used in machine learning (Ambroise et al., 2001; Grandvallet, 2002; Thiel et al., 2007; Schwenker and Trentin, 2014). Some methods consider these to be imprecise versions of a crisp (i.e., non-probabilistic) ground truth label, which they then try to estimate, while others (Thiel et al., 2007; Schwenker and Trentin, 2014), more in line with our setting, assume that probabilistic labels represent intrinsic ambiguity in class membership and consider them as ground truth. Methods such as *k*-nn (El Gayar et al., 2006) and support vector machines (Thiel et al., 2007; Scherer et al., 2013) have been adapted to deal with probabilistic labels, while regression-based methods, such as multi-layer perceptrons, can handle them without being adapted (Schwenker and Trentin, 2014). Yet, all of these methods are aimed at predicting a single class variable.

#### 2.7.3. Multi-dimensional classification

Multi-dimensional classification is more general than the related multi-label classification, which has already been considered in neuroscience (Turner et al., 2013). It is hard because the number of possible assignments to the class variables is exponential in their number. Predicting each class variable with an independent model is suboptimal because the variables are, generally, correlated. Modeling many of these dependencies, on the other hand, can lead to data scarcity. Multi-dimensional Bayesian network classifiers (Bielza et al., 2011; Borchani et al., 2013) can balance model complexity and the modeling of dependencies. However, they require crisp class labels in order to be trained and thus cannot be directly applied to our setting.

#### 2.7.4. K-nearest neighbors

*k* nearest neighbors is a popular instance-based (i.e., model-less) classifier. Among other extensions, the original *k*-nn classifier has been adapted to weight the effect of a neighbor's class label in proportion to how close that neighbor is to the data instance being classified (e.g., Dudani, 1976; MacLeod et al., 1987; Denoeux, 1995; Yazdani et al., 2009). It has also been adapted to deal with non-crisp labels (Jóźwik, 1983; Keller et al., 1985; Denoeux, 1995); these non-crisp labels, however, are not probabilistic but possibilistic (encoded with Dempster-Shafer theory) and fuzzy. Besides the handling of non-crisp labels, methods due to Denoeux (1995) and Keller et al. (1985) are similar to ours in that they weigh the neighbors' effect on prediction according to their closeness to the data instance being classified. On the other hand, they differ from our method in neither using probabilistic labels nor tackling the prediction of multiple class variables.

### 2.8. Experimental setting

We identified the nearest interneurons by measuring Euclidean distance. Thus, for a pair of interneurons **x**^*j*^ and **x**^*o*^, the distance *d*_*jo*_ is given by

$$d_{jo}={\left(\sum_{i=1}^{m}{\left(x_{i}^{\left(j\right)}-x_{i}^{\left(o\right)}\right)}^{2}\right)}^{\frac{1}{2}}.$$

Prior to computing distances, we standardized all predictor variables *X*_1_, …, *X*_*m*_ (i.e., for each *X*_*i*_, we subtracted its mean and divided by standard deviation).

We drew samples from the neighboring networks using probabilistic logic sampling (Henrion, 1986). We sought to draw enough samples from each distribution so to represent it correctly. We therefore set *M*, the total number of samples drawn from the *k* nearest neighbors' distributions (see Section 2.6.1), as *k* ∗ 500 ∗ *c*, where *c* was the maximal number of free parameters among the *k* networks whose consensus is being sought. The number of free parameters of a Bayesian network is the number of parameters that suffice to fully specify the network's probability distribution (recall that a network consists of a structure, 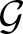, and parameters Θ; see subsection 2.5).

Once we had generated the data set of sample points, we applied a Bayesian network learning algorithm to obtain the consensus probability distribution.

#### 2.8.1. Learning Bayesian networks from data

There were two instances in which we learned Bayesian networks from data: when learning LBNs from expert-provided class label matrices (see Subsection 2.5) and when learning the consensus network from sampled data points (Subsection 2.6.1). We considered three options for the learning procedure and chose the one that we considered most adequate for learning LBNs, according to the criterion described in Subsection 2.5. We then applied this chosen procedure in both instances of network learning.

The Bayesian network learning procedure we used follows the well-known search+score approach. Such a procedure consists of (a) a search procedure for traversing the space of possible network structures and (b) a scoring function. We searched the structure space with the tabu metaheuristic (Glover, 1989, 1990), a local search procedure which employs adaptive memory to improve efficiency and escape local minima, and considered three networks scores: Bayesian Information Criterion (BIC; Schwarz, 1978), K2 (Cooper and Herskovits, 1992) and Bayesian Dirichlet equivalence (BDe; Heckerman et al., 1995). We compared the LBNs produced by the different scores according to how well they approximated the empirical distributions, *p*_ϵ_, (see Subsection 2.5) and their complexity (i.e., number of free parameters).

We estimated parameters by maximum likelihood estimation.

#### 2.8.2. Software and assessment

We implemented the computation of the 13 here introduced axonal morphometric parameters from scratch. We performed Bayesian network learning and sampling with the bnlearn (Scutari, 2010; Nagarajan et al., 2013) package for the R statistical software environment (R Core Team, 2014).

In traditional uni-dimensional classification, it is common to perform stratified cross-validation, that is, to have similar class proportions in train and test sets. However, such stratification is problematic in the multi-dimensional setting, due to the high number of combinations of class variables. Therefore, instead of stratified cross-validation, we evaluated our model with 20 repetitions of plain (unstratified) 10-fold cross-validation.

### 2.9. Assessing results

We were primarily interested in predicting LBNs. We assessed this prediction with Jensen-Shannon divergence, a metric which we describe below.

However, for comparison with related work on interneuron classification, we also assessed how well our method predicted crisp (i.e., non-probabilistic) labels. Such an evaluation is negatively biased against our method since we take label ambiguity into account to learn the model while it is evaluated as though a true crisp label existed (i.e., as if there was no ambiguity). Below we describe how we obtained crisp labels and present accuracy metrics for multi-dimensional classification.

#### 2.9.1. Comparing probability distributions

We measured the dissimilarity between two probability distributions, say *p*_
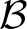
^(*u*)^_ and p_
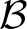
^*(*u*)^_, with Jensen-Shannon divergence,

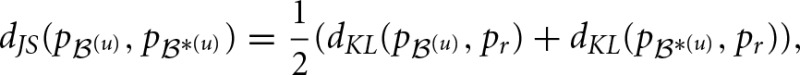



where 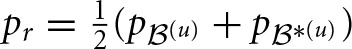 and *d*_*KL*_(*p*_
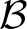
^(*u*)^_, p_
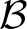
^*(*u*)^_) is the Kullback-Leibler divergence (Kullback and Leibler, 1951) between *p*_
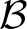
^(*u*)^_ and p_
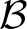
^*(*u*)^_,

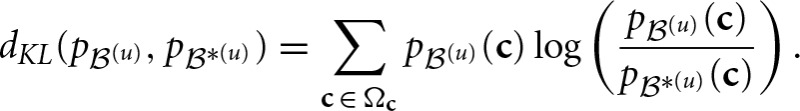



Unlike Kullback-Leibler divergence, Jensen-Shannon divergence is symmetric, it does not require absolute continuity (i.e., that p_
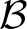
^*(*u*)^_(**c**) = 0 ⇒ *p*_
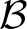
^(*u*)^_(**c**) = 0), its square root is a metric, and it is bounded: 0 ≤ *d*_*JS*_ ≤ 1.

#### 2.9.2. Obtaining crisp labels

In order to assess the prediction of crisp labels, we needed to obtain a “true” crisp class label vector for each interneuron **x**^(*j*)^. We assumed that such “true” labels were given by the choice of the majority of the experts. There were two alternative majority choices: (a) the most commonly selected class label vector, i.e., the most common row in a class labels matrix 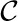; and (b) the concatenation of per-class majority labels, i.e., the vector formed by the most common choice for *C*_1_, the most common choice for *C*_2_, and so on, until *C*_5_. We refer to the former as the *joint truth* and to the latter as *marginal truth*; the latter was used in related works on interneuron classification (DeFelipe et al., 2013; Mihaljević et al., 2014a,b) since they predicted the axonal features *C*_1_–*C*_5_ independently. We compared our predicted crisp labels to both “truths.”

We also needed to extract crisp predictions from a predicted LBNs. The two straightforward methods are analogous to the above-described ones: (a) choosing the *most probable explanation* (MPE), i.e., the most likely joint assignment to **C** according to LBN 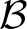
^*^); and (b) concatenating the marginally most likely assignments to each of the class variables. For simplicity, we only used the MPE as the predicted crisp class labels vector.

#### 2.9.3. Multi-dimensional classification accuracy metrics

We assessed crisp labels prediction with accuracy metrics for multi-dimensional classification (Bielza et al., 2011):

1. The *mean accuracy* over *d* (*d* = 5 in our case) class variables:
  $$\bar{Acc}=\frac{1}{d}\sum_{l=1}^{d}\frac{1}{N}\sum_{u=1}^{N}\delta \left(c_{l}^{*(u)},c_{l}^{\left(u\right)}\right),$$
  where c^*(*u*)^_l_ is the predicted value of *C*_l_ for *u*-th instance, *c*^(*u*)^_*l*_ is the corresponding true value, and δ(*a*, *b*) = 1 when *a* = *b* and 0 otherwise.
2. The *global accuracy* over *d* class variables:
  $$Acc=\frac{1}{N}\sum_{u=1}^{N}\delta \left(c^{*(u)},c^{\left(u\right)}\right)\cdot$$

Note that global accuracy is demanding as it only rewards full matches between the predicted vector and the true one. We also measured uni-dimensional *marginal accuracy* per each class variable,

$$Acc_{l}=\frac{1}{N}\sum_{u=1}^{N}\delta (c_{l}^{*(u)},c_{l}^{\left(u\right)}).$$

When computing global and mean accuracy, we used the “joint truth” crisp labels. When computing per-class-variable marginal accuracy, we used the “marginal truth” crisp labels vector.

## 3. Results

### 3.1. From multi-annotator labels to label Bayesian networks

We first studied whether any network score was particularly adequate for transforming multi-expert labels into LBNs. Different scores yielded networks of different degrees of complexity but were all good at approximating of the empirical probability distribution over the expert-provided labels, *p*_ϵ_ (see Table 1). We used the score that yielded the best approximation, BDe, in the remainder of this paper. Namely, we used it to (a) transform multi-expert labels into LBNs; and (b) learn a consensus networks from the generated samples.

**Table 1:** **Transforming multi-expert labels into label Bayesian networks using different network scores**.

	**BIC**	**K2**	**BDe**
JS divergence	00.10 ± 0.05	00.07 ± 0.04	00.06 ± 00.04
Free parameters	18.22 ± 1.83	31.08 ± 20.58	60.34 ± 31.14

Upper row: average Jensen-Shannon (JS) divergence between the empirical probability distribution over the labels, *p*_ϵ_, and the one encoded by the learned Bayesian network labels, *p*_
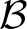
_; lower row: average number of free parameters per learned network. Averaged across entire data set.

### 3.2. Predicting label Bayesian networks

We considered four different values of *k* (the number of nearest neighbors)—namely, 3, 5, 7, and 9—, and obtained best results with *k* ∈ {5, 7}. As Table 2 shows, we predicted the label Bayesian networks relatively accurately, with a Jensen-Shannon divergence of 0.29 for *k* ∈ {5, 7}.

**Table 2:** **Predicting label Bayesian networks and crisp labels**.

	**JS**	**Global acc. (%)**	**Mean acc. (%)**
*k* = 3	0.30 ± 0.00	41.29 ± 1.57	79.10 ± 0.74
*k* = 5	0.29 ± 0.00	43.84 ± 1.48	79.52 ± 0.79
*k* = 7	0.29 ± 0.00	43.99 ± 1.26	79.88 ± 0.34
*k* = 9	0.30 ± 0.00	39.46 ± 1.67	78.58 ± 0.52

The leftmost column shows Jensen-Shannon (JS) divergence between predicted label Bayesian networks, p_
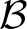
^*^_, and label Bayesian networks *p*_
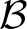
_ learned from 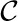. Rightmost columns show global and mean accuracy for predicting joint truth crisp labels vector, i.e., the class label vector most often selected by the experts. Obtained with 20 runs of 10-fold cross-validation.

Figure 2 depicts the true and predicted LBNs for two interneurons, one having barely ambiguous axonal features and another having an ambiguous type; as the figure suggests, the LBN of the former interneuron was accurately predicted, while in that of the latter, the type (*C*_5_) marginal probability was predicted only moderately well.

### 3.3. Predicting crisp labels

We predicted the joint truth (the class label vectors selected by a majority of experts; see Section 2.9.2) relatively accurately—with a mean accuracy of 80% and global accuracy of 44% for *k* ∈ {5, 7} (see Table 2). The latter result means that 44% of the MPEs of the predicted LBNs (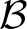
^*^) were equivalent to the joint truth vectors.

We also assessed the marginal accuracy for each axonal feature *C*_1_–*C*_5_. Here we compared the 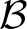
^*^ MPE with the marginal truth, class variable by class variable. We predicted features *C*_1_–*C*_4_ with over 80% accuracy—up to 88% in case of *C*_1_— and feature *C*_5_ with 64% accuracy with *k* = 7 (see Table 3). Albeit it may seem low, the latter result is better than chance. Namely, DeFelipe et al. (2013) showed that even 40.25% accuracy for *C*_5_—obtained by a classifier they used— was better than chance. It should also be recalled that the ten neuronal types were often hard to distinguish for expert neuroscientists (DeFelipe et al., 2013). Regarding the prediction of the individual types, accurately predicted ones included the MA and HT types, which were easy to identify for the experts, and the numerous but less clear to the experts types such as CB and LB. The least clear out of the numerous types, CT, was predicted with relatively low accuracy (see Table 4).

**Table 3:** **Accuracy (in %) for each of the five axonal features *C*_1_–*C*_5_**.

	***C***_**1**_	***C***_**2**_	***C***_**3**_	***C***_**4**_	***C***_**5**_
*k* = 3	86.15 ± 1.12	83.17 ± 0.98	86.50 ± 0.88	83.11 ± 0.90	62.69 ± 1.24
*k* = 5	86.49 ± 0.98	83.25 ± 0.79	86.05 ± 0.79	84.18 ± 0.65	63.78 ± 1.11
*k* = 7	88.07 ± 1.01	83.12 ± 0.72	85.29 ± 0.55	84.06 ± 0.74	64.33 ± 1.52
*k* = 9	87.16 ± 1.03	83.06 ± 0.78	85.39 ± 0.78	83.88 ± 0.71	63.79 ± 1.59

Here we compared the marginal true labels to the most probable explanation of the predicted label Bayesian networks. Obtained with 20 runs of 10-fold cross-validation.

**Table 4:** **Confusion matrix for predicting *C*_5_ with *k* = 7**.

	**CB**	**CH**	**CT**	**HT**	**LB**	**MA**	**NG**	**Per-type sensitivity**
CB	41	0	10	0	6	3	0	0.68
CH	2	0	1	0	0	0	0	0.00
CT	10	0	25	4	8	11	0	0.43
HT	0	0	3	10	1	0	0	0.71
LB	9	0	3	0	25	3	1	0.61
MA	0	0	2	0	4	36	0	0.86
NG	8	0	0	0	0	0	0	0.00

Here we compared the marginal true label for *C*_5_ (rows) to the *C*_5_ value of most probable explanation of the predicted label Bayesian network (columns). The rightmost column shows per-type sensitivity. Types AR, and CR and OT are omitted since no cell's crisp label was of one of these types. Obtained from a single run of 10-fold cross-validation.

## 4. Discussion

Previous studies on interneuron classification (DeFelipe et al., 2013; Mihaljević et al., 2014a,b) used majority crisp labels, estimated for each axonal feature independently, to train and evaluate their models. Mihaljević et al. (2014a) only considered *C*_5_ whereas DeFelipe et al. (2013) and Mihaljević et al. (2014a) predicted axonal features *C*_1_–*C*_5_ with an independent model for each of them. There were non-methodological differences among these two studies and the present work and therefore any comparison of results ought to be performed with some caution. DeFelipe et al. (2013), for example, used 15 cells more than we did (see Section 2.4), had several of variables' values corrupted by imperfections in the reconstructions of 36 cells—which we corrected—, and used only three values for *C*_4_—ascending, descending, and both. Furthermore, they used different morphometric predictor parameters (over 2000 of them), and applied a possibly more optimistic accuracy estimation technique—leave-one-out estimation. Mihaljević et al. (2014a) considered multiple subsets of the data, formed according to the degree of class label ambiguity of the included cells, and obtained best results with least ambiguous cells (e.g., with 46 cells for *C*_5_). Their best results were thus obtained with a small subset of the 226 cells that we used. When using most of the cells, their results were similar to those of DeFelipe et al. (2013).

Differences aside, in Table 5 we compare the accuracies from the present study with those from DeFelipe et al. (2013). We outperformed DeFelipe et al. (2013) in predictive accuracy for every axonal feature, even though we used a single model to predict all features simultaneously. We especially outperformed their approach in predicting *C*_3_ and, even more, in predicting *C*_4_. The latter was likely affected by the use of the additional category no (see subsection 2.2).

**Table 5:** **Our best predictive accuracy (in %) vs. best accuracy from DeFelipe et al. (2013), for each of the axonal features *C*_1_–*C*_5_**.

	***C***_**1**_	***C***_2_	***C***_**3**_	***C***_**4**_	***C***_**5**_
Present study	88.07	83.25	86.50	84.18	64.33
DeFelipe et al., 2013	85.48	81.33	73.86	60.17	62.24

Our results were obtained with 20 runs of 10-fold cross-validation; those of DeFelipe et al., (2013) were obtained with leave-one-out cross-validation.

Despite the non-methodological differences with the study by DeFelipe et al. (2013), the better accuracies that we achieved might suggest some or all of the following: (a) the introduced morphometric parameters are useful for predicting interneuron type and axonal features; (b) we adequately assigned the value no for cells to which the other values of *C*_4_ did not apply; and (c) our method is adequate for classifying interneurons.

The above results, along with the relatively high global accuracy achieved, 44%, suggest that axonal features *C*_1_–*C*_5_ are interrelated and that it is useful to attempt predicting them simultaneously.

Finally, several other efforts regarding classification of neurons in general have been performed taking into account other morphological and/or molecular and/or electrophysiological properties (e.g., Bota and Swanson, 2007; Ascoli et al., 2009; Brown and Hestrin, 2009; Battaglia et al., 2013; Sümbül et al., 2014). These studies indicate that in spite of a large diversity of neuronal types, certain clear correlations exist between the axonal features and dendritic morphologies, and between these anatomical characteristics and some molecular and electrical attributes. Nevertheless, the classification of neurons is still under intense study from different angles, including anatomical, physiological, and molecular criteria, and using a variety of mathematical approaches, such as hierarchical clustering (Cauli et al., 2000; Wang et al., 2002; Tsiola et al., 2003; Benavides-Piccione et al., 2006; Dumitriu et al., 2007; Helmstaedter et al., 2009a,b), *k*-means (e.g., Karagiannis et al., 2009, affinity propagation (Santana et al., 2013), linear discriminant analysis (Marin et al., 2002; Druckmann et al., 2013), Bayesian network classifiers (Mihaljević et al., 2014a), and semi-supervised model-based clustering (Mihaljević et al., 2014b).

### 4.1. Computing axonal morphometric parameters

In order to compute some of the newly introduced axonal morphometric parameters—namely, those relative to laminar and cortical distribution—, we followed a series of assumptions originating from DeFelipe et al. (2013). These assumptions (simplifications) should be kept in mind when interpreting our results. First, we assumed arbitrary columnar and laminar demarcations. Namely, we considered the diameter of the hypothetical cortical column to be 300 μm (Malach, 1994; Mountcastle, 1998), whereas laminar thickness was estimated for each neuron from its original paper, when such a paper was available, and from relevant literature otherwise. Finally, we assumed that a soma is always located in the center of its layer and cortical column.

## 5. Conclusion

We built a model that can automatically classify an interneuron, on the basis of a set of its axonal morphometric parameters, according to five properties which constitute the pragmatic classification scheme proposed by DeFelipe et al. (2013), namely, the interneuron type and four other categorical axonal features. We guided model construction with a Bayesian network-encoded probability distribution indicating the type and axonal features of each interneuron. We obtained these probability distributions from classification choices provided by a group of leading neuroscientists. We then developed an instance-based supervised classifier which could learn from such multi-dimensional probabilistic input, predicting the output by forming a consensus among a set of Bayesian networks.

We accurately predicted the probabilistic labels over the interneuron type and the four remaining axonal features. Furthermore, we outperformed previous work when predicting crisp (i.e., non-probabilistic) labels. Importantly, and unlike previous work, we predicted the five axonal features simultaneously (i.e., with a single model), which is useful since these features are complementary. Our results suggest that interneuron type and the and four remaining axonal features are related and that it is useful to predict them jointly.

We introduced 13 axonal morphometric parameters which we defined as quantitative counterparts of the four categorical axonal features. Our results suggest that these parameters are useful for predicting the type and the four axonal features. Thus, they might be considered as objective replacements, or surrogates, of the subjective categorical axonal features.

This paper demonstrates a useful application of Bayesian networks in neuroscience, whose potential has been largely unexploited in this field (one exception is functional connectivity analysis from neuroimaging data; see Bielza and Larrañaga, 2014).

It would be interesting to relax the assumption that all neuroscientists who classified our data are equally accurate at classifying all types of interneurons, since some may be more familiar with certain interneuron types than with others, and account for expert competence in our model, similarly to methods for learning from a crowd of annotators such as Raykar et al. (2010) and Welinder et al. (2010).

We also intend to consider new methods for forming a consensus among Bayesian networks.

### 5.1. Data sharing

The data set and the software reproducing our study are available online, at http://cig.fi.upm.es/bojan/gardener/.

## Author contributions

Bojan Mihaljević, Concha Bielza, and Pedro Larrañaga designed the method and the empirical study. Ruth Benavides-Piccione corrected the faulty interneuron reconstructions. Ruth Benavides-Piccione and Bojan Mihaljević defined the here introduced morphological variables. Bojan Mihaljević performed the data analysis, implementing necessary software, and wrote the paper. Concha Bielza, Ruth Benavides-Piccione, Javier DeFelipe and Pedro Larrañaga critically revised the paper.

### Conflict of interest statement

The authors declare that the research was conducted in the absence of any commercial or financial relationships that could be construed as a potential conflict of interest.

## Footnotes

^1^Following DeFelipe et al. (2013), we will often refer to the type and the four axonal features simply as *axonal features* (i.e., interneuron type is also encompassed by this term).

^2^From this point on, we adopt some machine learning terminology. Namely, in machine learning, each (discrete) predicted variable—in our case, these are the five axonal features— is called a *class variable*; this term, therefore, applies to each of the five axonal features, even though only the neuronal type is a class in the usual meaning of this term in neuroscience. *Class labels* are the assignments to the class variables associated with the data points (interneurons); e.g., an interneuron might be labeled as intralaminar. We will sometimes refer to expert neuroscientists as *annotators* because they annotated (labeled) the data with class labels. *Predictor variables* are the independent variables in a model, e.g., we use morphometric properties of an interneuron as predictor variables to predict interneuron type and other axonal features (the latter are the class variables). We will not use the term “features” as a synonym of “predictor variables,” as commonly done in machine learning, in order to avoid confusion with the term “axonal features.” We will use the terms “axonal features” and “class variables” interchangeably.

^3^Only neuroscientists who considered that **x**^(*j*)^ was characterized (axonal feature *C*_6_) labeled **x**^(*j*)^ according to *C*_1_–*C*_5_. Therefore, *N*_*j*_ may be less than 42 and varies across interneurons.

^4^Possibly, the ground truth might be better approximated by consulting more leading neuroscientists. Our group, nonetheless, includes many of the leading experts involved in the interneuron nomenclature effort (Ascoli et al., 2007).
